# Reduced speech coherence in psychosis-related social media forum posts

**DOI:** 10.1038/s41537-024-00481-1

**Published:** 2024-07-04

**Authors:** Laurin Plank, Armin Zlomuzica

**Affiliations:** Department of Behavioral and Clinical Neuroscience, Ruhr-University Bochum (RUB), D-44787 Bochum, Germany

**Keywords:** Psychosis, Human behaviour

## Abstract

The extraction of linguistic markers from social media posts, which are indicative of the onset and course of mental disorders, offers great potential for mental healthcare. In the present study, we extracted over one million posts from the popular social media platform Reddit to analyze speech coherence, which reflects formal thought disorder and is a characteristic feature of schizophrenia and associated psychotic disorders. Natural language processing (NLP) models were used to perform an automated quantification of speech coherence. We could demonstrate that users who are active on forums geared towards disorders with a higher degree of psychotic symptoms tend to show a lower level of coherence. The lowest coherence scores were found in users of forums on dissociative identity disorder, schizophrenia, and bipolar disorder. In contrast, a relatively high level of coherence was detected in users of forums related to obsessive–compulsive disorder, anxiety, and depression. Users of forums on posttraumatic stress disorder, autism, and attention-deficit hyperactivity disorder exhibited medium-level coherence. Our findings provide promising first evidence for the possible utility of NLP-based coherence analyses for the early detection and prevention of psychosis on the basis of posts gathered from publicly available social media data. This opens new avenues for large-scale prevention programs aimed at high-risk populations.

## Introduction

Evidence from large epidemiological studies suggests an underrecognition and undertreatment of common mental disorders^1^. Additionally, access to mental healthcare around the world is severely limited, and individuals suffering from mental illness often fail to receive adequate treatment^2^. As a consequence, individuals suffering from mental health problems increasingly turn to social media to share their personal experiences, seek information, and receive peer support^2,3^. Reddit is among the most widely used social media platforms with over 267 million unique active users each week^4^. On Reddit, users interact with other users by posting or commenting on other people’s posts. By subscribing to forums (“subreddits”) related to mental health such as “r/depression” and “r/anxiety”, users may share their personal experiences with a mental disorder or receive support from peers suffering from the same condition.

Over the past years, analyzing data gathered from Reddit posts has become a powerful tool to gain novel insight into common mental health disorders^5,6^. Data derived from social media may be used to identify individuals who potentially suffer from mental health problems or to discern the effects of mental disorders on real-life behavior^7,8^. Therefore, analyzing data from social media data bears great potential to overcome the underrecognition and undertreatment of mental disorders^9^. The biggest advantage of analyzing social media posts is that they reflect unconstrained behavior under real-life circumstances. This enables the collection of data with a high degree of ecological validity, much unlike data obtained in laboratory conditions^10^.

Posts from social media forums such as Reddit can be analyzed efficiently by means of natural language processing (NLP)^11^ to understand processes related to substance abuse^12^, anxiety and depression^5,13,14^, suicidality^15^ or bipolar disorder (BD)^16^. Social media data has also been utilized in the study of psychosis and schizophrenia (SZ)^8,17–27^. Posts made by individuals suspected of suffering from psychosis were used to evaluate the nature and impact of COVID-19 on subjective well-being^24^ or to study their sleep behavior^20^. Importantly, linguistic features from these posts have been used to predict whether a user is likely or actually suffering from psychosis^17–19,23,25,26^ and whether a symptom relapse is likely to occur^18^. The ultimate goal of these studies might be the development of systems for the automated and remote prediction, diagnosis, and monitoring of psychosis. Since roughly 5 billion people use social media worldwide^28^, the analysis of online behavior could allow mental healthcare to reach more people, especially those who have otherwise no access to it.

Disorganized speech is a prevalent characteristic among individuals diagnosed with psychotic disorders such as SZ. This symptom reflects one important domain of formal thought disorder (FTD), which is a key symptom of SZ, and can be derived from the analysis of language^29^ produced in the context of real-life narratives^30^. One specific aspect of disorganized speech, termed “speech coherence”, refers to the flow in the meaning in sentences^31^. Speech coherence can be measured using computational methods^32,33^. The computational measurement of coherence corresponds mainly to aspects of positive thought disorder, namely tangentiality and derailment of speech^31^, which reflect a “loosening of associations”^34,35^. This dimension of speech impairment is characteristic of schizophrenia and other psychotic disorders^34^ and thus can be used to determine whether psychosis-related symptomatology is present within a certain population.

Multiple studies have tested whether the automated assessment of speech coherence may be used to detect SZ. The results suggest a lower coherence in patients suffering from or at risk of SZ when compared to healthy controls^32,36–45^ although results are not entirely consistent^33,42,46^. Importantly, speech coherence was also found to predict an SZ diagnosis and positive and negative symptoms with a good accuracy month in advance^37,38^. These findings collectively indicate that the use of NLP for analysis of speech coherence offers great potential to detect subjects who are at risk of developing psychosis, as well as to inform effective prevention and treatment regimens.

Surprisingly, no study has thus far analyzed the coherence of social media posts made by users who indicate that they suffer from psychotic symptoms. Hence, the aim of the present study was to examine whether reduced speech coherence typically found in patients suffering from SZ might also be evidenced in posts made in online forums for individuals suffering from psychosis. We first investigate whether coherence may be reduced in a forum on SZ, as only SZ reductions in coherence have been established using computational methods. As the control group, a forum on depression (r/depression) was considered. Mental health-related forums represent a more appropriate control group than non-mental health-related forums. The content of posts made in mental health-related forums and the users’ characteristics are likely more comparable. Furthermore, SZ and depression share a common negative symptomatology while FTD symptoms are more prevalent in SZ^47^. We expected that the coherence of posts made in r/schizophrenia would be lower than the coherence of posts made in r/depression.

We further analyzed coherence from posts in seven other subreddits on mental health (posttraumatic stress disorder (PTSD), attention deficit hyperactivity disorder (ADHD), anxiety, obsessive–compulsive disorder (OCD), autism, BD, dissociative identity disorder (DID)). Varying levels of psychotic symptoms have been found in these disorders. Psychotic symptoms are relatively well documented and most pronounced in BD^48–50^ and DID^51,52^. For PTSD^53^ and autism^54^ evidence for associations with psychotic symptoms has also been described, although to a lesser extent. While associations between psychotic symptoms and OCD, ADHD, anxiety disorders, and depression have been reported^55–58^, they seem to be less pronounced as compared to BD and DID. Thus, reduced speech coherence is typically found in SZ patients but might also be evidenced in individuals with psychotic symptoms having another primary diagnosis^59–61^. We therefore examined whether speech coherence varies across subreddits dedicated to mental health disorders which, to a greater or lesser extent, are associated with psychotic symptoms. We expected that higher rates of psychotic symptoms would coincide with lower coherence scores for the respective disorder categories. This type of analysis could inform more elaborated laboratory-based studies on speech coherence in clinically diagnosed participants suffering from various mental disorders.

As a reference for the coherence observed in the general population on Reddit, five popular non-mental health-related subreddits were chosen as a control group. The subreddits “r/self”, “r/relationship_advice”, “r/dating_advice”, “r/pettyrevenge” and “r/socialskills” were selected, because they contain posts similar in format to the mental health subreddit posts (mostly text paragraphs describing personal experience).

It is possible that discovered patterns of speech coherence are specific to posts made in mental health subreddits. One reason for this may be that users adapt their writing to other posts that they are reading, which could skew their writing further toward or away from coherence. To test whether such processes could influence the results, a second dataset comprising posts made in non-mental health-related subreddits was extracted.

## Methods

As this study analyzed publicly available data, the approval of an ethics committee was not required. Data extraction, filtration, and preprocessing were performed in Python.

### Data extraction

Posts were downloaded from a repository of Reddit submissions created by the Pushshift project^62^. Data provided by the Pushshift project has been extensively used in prior research^6^.

#### Dataset 1

All Reddit submissions made in the 36 months between the 1st of January 2021 and the 31st of December 2023 were downloaded. Subsequently, all submissions from the subreddits “r/Anxiety”, “r/OCD”, ‘r/depression”, ‘r/ADHD”, “r/autism”, “r/schizophrenia”, “r/ptsd”, “r/bipolar”, “r/DID” were extracted. In sum, 1,920,933 posts were extracted. Additionally, 300,000 non-deleted posts were randomly sampled from the control group. We refer to this dataset as dataset 1.

#### Dataset 2

The extraction of posts by control users and mental health-subreddit users in non-mental health-related subreddits followed a similar approach to Robertson et al.^63^. For each month in the year 2023, users submitting to each subreddit were extracted. If more than 1000 users were extracted, 1000 users were randomly sampled. Then, for the given month, all posts made by these users were extracted. Only posts that were not removed, deleted or empty were considered. Furthermore, posts made in popular mental health subreddits or the control forums were excluded. We refer to this dataset as dataset 2. In both datasets, only those posts starting a new thread (not comments to those posts) and only the body of text (not its header) were included in the analysis.

### Data filtration

The presence of URLs was determined using the “urlextract” package and posts were discarded if they contained URLs. The “textblob” package, which is built on the “nltk” package, was used to tokenize the posts’ texts (divided into sentences). The function “.sentences” applied to a “TextBlob” object divides texts based on punctuation patterns but respects special cases, such as within-sentence punctuation-use (e.g., “Dr.”). Only posts that contained at least two sentences were submitted to preprocessing. Posts containing media content were only included if they also contained written text that conformed to the aforementioned criteria. The media content itself was ignored in those cases.

### Data preprocessing

**Fig. 1:**
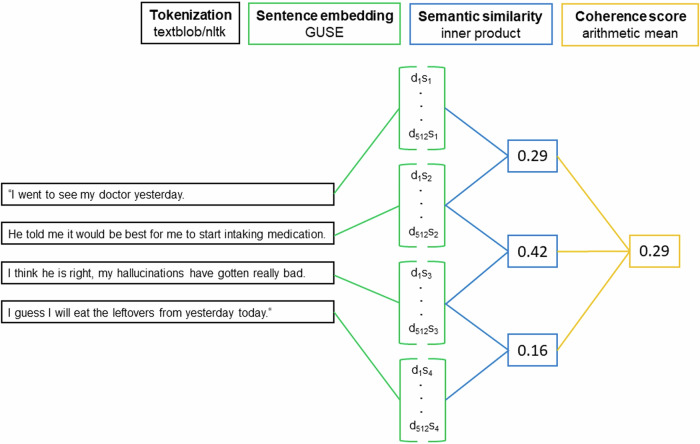
Coherence calculation process. *Note*. Paragraphs were first divided into sentences (tokenization). Afterward, sentences were embedded into 512-dimensional semantic space using Google’s Universal Sentence Encoder (GUSE) (Cer et al., 2018). The inner product of the embeddings of subsequent sentences represents their semantic similarity. The average of all sentence pairs’ similarity yielded the coherence scores. d_1_s_1_ denotes the first embedding dimension of the embedding vector of the first sentence. The narrative is quite coherent until the last sentence pair which is marked by a lower semantic similarity and thus represents a break in coherence.

Like previous studies^31–33^, we operationalize coherence as the average semantic similarity between subsequent sentences. Coherence scores were calculated by first embedding all sentences of a post into 512-dimensional semantic space using GUSE^64^. Afterward, the inner product between the embeddings of subsequent sentences was calculated and averaged for each post^32,33^. The inner product of two sentence embeddings, which ranges from −1 (low) to 1 (high), represents their semantic similarity. Our approach to the calculation of coherence is similar to previous studies such as Iter et al.^32^ and Just et al.^33^. A graphical illustration of the coherence calculation process is given in Fig. 1.

Reddit forums impose idiosyncratic rules as to the content that may be posted. These rules are enforced through the deletion of posts that stand in violation of said rules. For example, in r/depression, users are explicitly asked to refrain from posting uplifting content, while no such rule exists in r/schizophrenia. We therefore deemed it necessary to control for other textual features, such as the posts’ emotional tone, that might systematically influence coherence scores. To this end, a sentiment analysis was performed using the “textblob” package. The sentiment analysis yields two values. The polarity, which will be referred to as emotional valence, is a measure ranging from −1 (negative) to 1 (positive) which captures the emotional tone of the texts. The subjectivity ranges from 0 (objective) to 1 (subjective) and captures the degree to which the text refers to personal experiences instead of impersonal facts.

### Statistical analysis

The statistical analysis and plot creation were performed in R (R Core Team, 2013). Two-sided t-tests were used to test for differences between r/depression and r/schizophrenia on the covariates. Regression analyses were used to test for differences between groups on the coherence measure while controlling for the covariates. Lastly, post hoc pairwise comparisons were performed on the estimated marginal means from the regression models. To account for multiple tests, *p*-values were corrected using Tukey’s method. Statistical tests were considered significant at *p*-values below 0.05. Due to very small *p*-values and standard error estimates caused by large sample sizes, reported effect sizes should be taken into consideration.

## Results

After data filtration dataset 1 consisted of 1,310,154 posts. Characteristics of the posts made in the different subreddits and the control group are shown in Table 1.

**Table 1:** Characteristics of the posts made in the different subreddits.

Subreddit	Members	*N*_pre-filter_	*N*_post-filter_	Words: M[SD]	Sentences: M[SD]
depression	1,010,954	492,710	242,233	204.24 [221.21]	11.83 [13.01]
Anxiety	664,712	269,624	165,118	173.72 [167.29]	9.51 [9.12]
OCD	208,556	167,096	102,018	183.66 [194.13]	9.4 [9.84]
ADHD	1,803,530	459,254	241,066	201.42 [167.54]	10.64 [9.07]
autism	343,339	229,875	120,842	180.43 [187.49]	9.29 [9.64]
ptsd	100,952	37,828	23,994	231.39 [255.9]	12.89 [14.54]
bipolar	218,384	152,244	73,646	161.73 [156.34]	9.58 [9.04]
schizophrenia	75,905	63,981	27,422	155.55 [182.76]	8.91 [9.79]
DID	60,796	48,321	28,750	210.79 [200.79]	11.54 [11.31]
Control group			285,065	327.78 [296.39]	16.25 [15.13]

*Note*. The member count was retrieved on 2024-03-05. The control group consisted of posts submitted to the subreddits “r/self”, “r/dating_advice”, ‘r/socialskills”, “r/relationship_advice” and “r/pettyrevenge”. The subreddits are popular (each has over one million members) and users post content similar in form to the mental health subreddits. The filtration steps included the deletion of posts that were empty, shorter than two sentences or contained a URL.

### Comparison of r/schizophrenia and r/depression

Independent sample *t*-tests were performed to test for differences in r/depression and r/schizophrenia on the confounding variables. Posts in r/schizophrenia [M = 155.55, SD = 221.21] contained significantly fewer words than posts in r/depression [M = 204.24, SD = 182.76], *t*(37154) = 40.85, *p* < 0.001, 95% CI = 46.35–51.02, *d* = 0.22 [95% CI = 0.21–0.24]. Furthermore, posts in r/schizophrenia [M = 8.9, SD = 9.79] contained significantly fewer sentences than posts made in r/depression [M = 11.83, SD = 13.01], *t*(39300) = 45.12, *p* < 0.001, 95% CI = 2.8–3.05, *d* = 0.23 [95% CI = 0.22–0.24]. The sentence length was significantly lower in r/schizophrenia [M = 17.96, SD = 12.02] than in r/depression [M = 19.13, SD = 14.36], *t*(36885) = 14.92, *p* < 0.001, 95% CI = 1.01–1.32, *d* = 0.08[95% CI = 0.07–0.1]. The posts’ emotional tone were significantly more positive in r/schizophrenia [M = 0.03, SD = 0.19], when compared to r/depression [M = 0.001, SD = 0.17], *t*(33034) = 25.65, *p* < 0.001, 95% CI = 0.028–0.032], *d* = 0.17[95% CI = 0.16–0.19]. Lastly, posts in r/schizophrenia [M = 0.49, SD = 0.17] showed significantly less reference to subjective experience than posts in r/depression [M = 0.52, SD = 0.14], *t*(31657) = 27.39, *p* < 0.001, 95% CI = 0.027–0.031, *d* = 0.21[95% CI = 0.19–0.22]. As all comparisons yielded significant effects, all confounding variables were included in subsequent analyses.

An ordinary least square (OLS) multiple regression was fit to the data with coherence as the criterion and subreddit (0 = r/depression vs. 1 = r/schizophrenia), word count, sentence count, sentence length, emotional tone, and subjectivity as predictors. The predictors significantly explained variations in coherence, adj. *R*^2^ = 0.099, *F*(6, 269648) = 4958, *p* < 0.001. There were significant effects of the predictors *subreddit*, *β* = −0.021, *t* = −39.79, *p* < 0.001, *η*^2^*p* = 0.006, *word count*, *β* = 0.00003, *t* = 14.23, *p* < 0.001, *η*^2^*p* = 0.0008, *sentence count*, *β* = −0.0009, *t* = −28.13, *p* < 0.001, *η*^2^*p* = 0.003, *sentence length*, *β* = 0.002, *t* = 111.66, p < 0.001, *η*^2^*p* = 0.044, *subjectivity*, *β* = 0.028, *t* = 23.3, *p* < 0.001, *η*^2^*p* = 0.002, and *emotional valence*, *β* = −0.037, *t* = −38.93, *p* < 0.001, *η*^2^*p* = 0.006. The estimated marginal mean of coherence was lower in posts made in r/schizophrenia [M = 0.251, SE = 0.0002] than in r/depression [M = 0.272, SE = 0.0005]. The comparison yielded a Cohen’s *d* of 0.255 [95% CI = 0.242–0.268]. A post-hoc power analysis suggested that the achieved power of this comparison closely approaches 1.

### Coherence across all subreddits

Next, the entire sample of subreddits was considered. An OLS multiple regression significantly explained variations in coherence, adj. *R*^2^ = 0.12, *F*(13,1025075) = 1077, *p* < 0.001. Again, the dummy-coded variable *subreddit* (0 = r/schizophrenia) significantly predicted the coherence scores while controlling for the confounding variables, all comparisons *p* < 0.001. The effects of the confounding variables were the same as in the previous regression model. Post hoc pairwise comparisons were performed on the estimated marginal means derived from the regression model. Multiple comparisons were adjusted using Tukey’s method. The results of the pairwise comparisons are listed in Table 2.

**Table 2:** Estimated marginal means for the coherence scores of all subreddits and significant post-hoc comparisons.

Subreddit	Coherence: M [SE]	Coherence sign. lower than	Adj. *p*-value	Effect size: Cohen’s *d* [95% CI]
Control	0.276 [0.0002]			
depression	0.275 [0.0002]			
		Control	0.026	0.01 [0.004–0.015]
Anxiety	0.272 [0.0002]			
		Control	<0.001	0.04 [0.034–0.047]
		depression	<0.001	0.031 [0.025–0.037]
OCD	0.268 [0.0003]			
		Control	<0.001	0.096 [0.088–0.103]
		depression	<0.001	0.086 [0.079–0.093]
		Anxiety	<0.001	0.055 [0.048–0.063]
bipolar	0.259 [0.0003]			
		Control	<0.001	0.2 [0.191–0.208]
		depression	<0.001	0.19 [0.182–0.198]
		Anxiety	<0.001	0.159 [0.151–0.168]
		OCD	<0.001	0.104 [0.095–0.114]
ADHD	0.259 [0.0002]			
		Control	<0.001	0.204 [0.198–0.209]
		depression	<0.001	0.194 [0.188–0.2]
		Anxiety	<0.001	0.163 [0.157–0.17]
		OCD	<0.001	0.108 [0.101–0.115]
ptsd	0.258 [0.0005]			
		Control	<0.001	0.214 [0.201–0.227]
		depression	<0.001	0.204 [0.191–0.218]
		Anxiety	<0.001	0.174 [0.16–0.187]
		OCD	<0.001	0.118 [0.104–0.132]
autism	0.255 [0.0002]			
		Control	<0.001	0.25 [0.243–0.256]
		depression	<0.001	0.24 [0.233–0.247]
		Anxiety	<0.001	0.209 [0.202–0.217]
		OCD	<0.001	0.154 [0.146–0.162]
		bipolar	<0.001	0.05 [0.041–0.059]
		ADHD	<0.001	0.046 [0.039–0.053]
		ptsd	<0.001	0.036 [0.022–0.049]
schizophrenia	0.253 [0.0005]			
		Control	<0.001	0.272 [0.259–0.284]
		depression	<0.001	0.262 [0.25–0.275]
		Anxiety	<0.001	0.232 [0.219–0.244]
		OCD	<0.001	0.176 [0.163–0.19]
		bipolar	<0.001	0.072 [0.058–0.086]
		ADHD	<0.001	0.068 [0.056–0.081]
		ptsd	<0.001	0.058 [0.041–0.075]
		autism	0.027	0.022 [0.009–0.036]
DID	0.23 [0.0005]			
		Control	<0.001	0.55 [0.538–0.563]
		depression	<0.001	0.541 [0.529–0.553]
		Anxiety	<0.001	0.51 [0.498–0.523]
		OCD	<0.001	0.455 [0.442–0.468]
		bipolar	<0.001	0.351 [0.337–0.364]
		ADHD	<0.001	0.347 [0.335–0.359]
		ptsd	<0.001	0.336 [0.319–0.354]
		autism	<0.001	0.301 [0.288–0.314]
		schizophrenia	<0.001	0.278 [0.262–0.295]

*Note*. Pairwise comparisons were performed on estimated marginal means, and multiple comparisons were adjusted for using Tukey’s method.

**Fig. 2:**
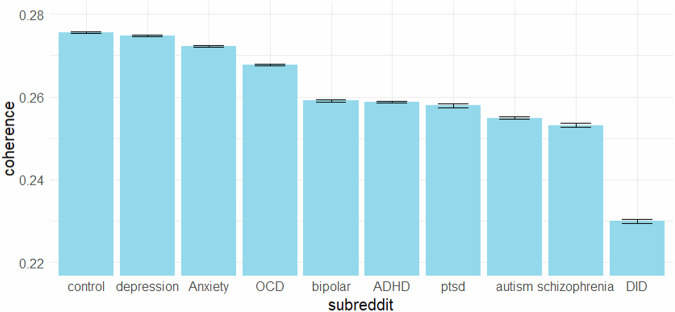
Estimated marginal means for the coherence scores of all ten subreddits. *Note*. Error bars represent the standard error of the estimated marginal means. The control group is composed of a sample of posts derived from the subreddits “r/self”, “r/relationship_advice”, “r/dating_advice”, “r/pettyrevenge” and “r/socialskills”.

The lowest level of coherence was found in r/DID, followed by r/schizophrenia and r/autism. Medium-level coherence scores were found in r/ptsd and r/ADHD and r/bipolar. Coherence scores were the highest in the control group, followed by r/depression, r/Anxiety, and r/OCD. The estimated marginal means for the coherence of all ten subreddits and the post-hoc comparisons are shown in Fig. 2 and Table 2.

### Generalization to non-mental health-related subreddits

**Fig. 3:**
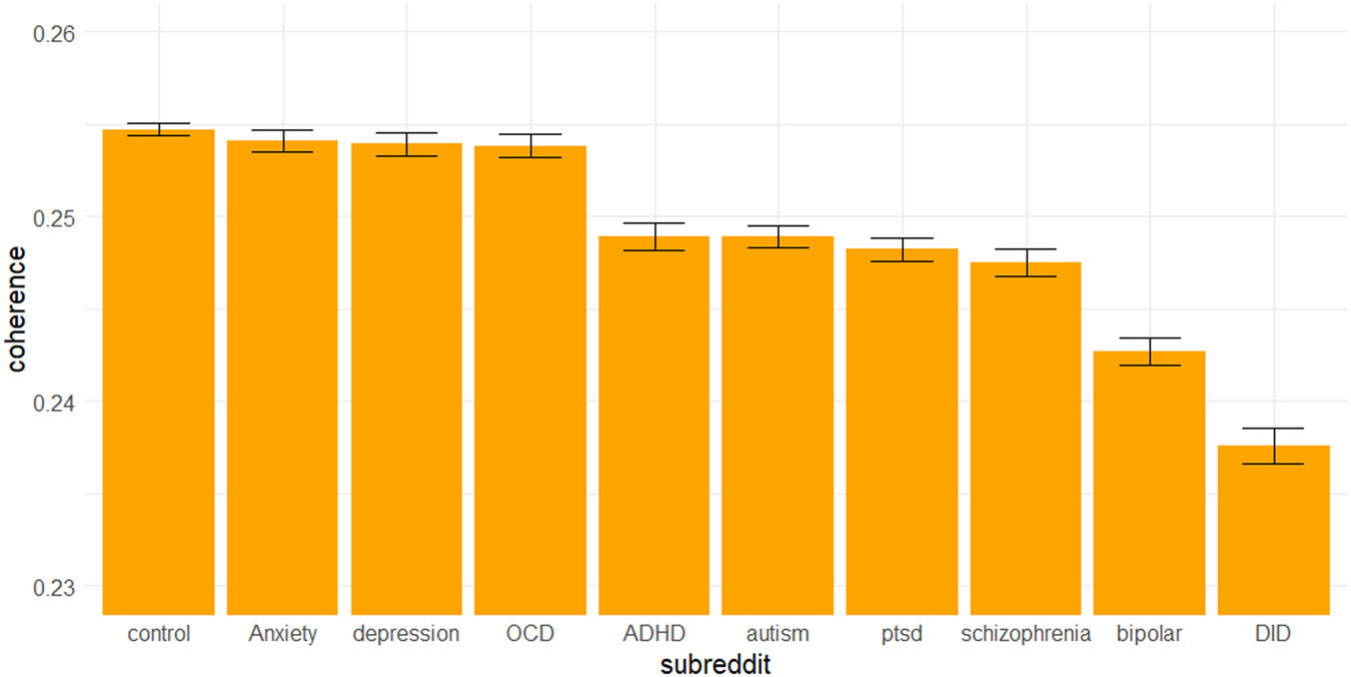
Estimated marginal means for the coherence scores of posts made in neutral subreddits. *Note*. Error bars represent the standard error of the estimated marginal means. The control group is composed of posts made by users of the subreddits “r/self”, “r/relationship_advice”, “r/dating_advice”, “r/pettyrevenge” and “r/socialskills”. The sample sizes after filtration were *N*_control_ = 121,897, *N*_Anxiety_ = 35,108, *N*_depression_ = 26,499, *N*_OCD_ = 30,208, *N*_ADHD_ = 22,967, *N*_autism_ = 30,818, *N*_ptsd_ = 30,533, *N*_schizophrenia_ = 23,026, *N*_bipolar_ = 20,152, *N*_DID_ = 12,405.

After data filtration, dataset 2 contained 353613 posts (*N*_control_ = 121,897, *N*_Anxiety_ = 35,108, *N*_depression_ = 26,499, *N*_OCD_ = 30,208, *N*_ADHD_ = 22,967, *N*_autism_ = 30,818, *N*_ptsd_ = 30,533, *N*_schizophrenia_ = 23,026, *N*_bipolar_ = 20,152, *N*_DID_ = 12,405). An OLS multiple regression was fit to the data with the same predictors and criterion as in the previous regressions. The regression model significantly explained variation in coherence scores, adj. *R*^2^ = 0.09, *F*(14,353598) = 2550, *p* < 0.001. All predictors significantly predicted the coherence scores. In contrast to the previous regression models, *subjectivity* now negatively predicted the coherence scores, *β* = −0.003, *t* = −23.45, *p* < 0.001, *η*^2^*p* = 0.002. All other regression coefficients retained their sign from the previous regressions. Based on this regression model, estimated marginal means were computed and used for pairwise comparisons. Multiple comparisons were adjusted for using Tukey’s method. Figure 3 and Table 3 depict the estimated marginal means, standard errors, and results of the pairwise comparisons. Coherence scores were the highest for the control group, r/Anxiety, r/depression, and r/OCD, and did not significantly differ from each other. Coherence scores were the lowest for r/bipolar and r/DID. Medium-level coherence scores emerged for r/ADHD, r/autism, r/ptsd, and r/schizophrenia, with no significant differences.

**Table 3:** Estimated marginal means for the coherence scores of posts made in neutral subreddits and significant post-hoc comparisons.

Subreddit	Coherence: M [SE]	Coherence sign. lower than	Adj. *p*-value	Effect size: Cohen’s *d* [95% CI]
Control	0.255 [0.0003]			
Anxiety	0.254 [0.0006]			
depression	0.254 [0.0007]			
OCD	0.254 [0.0006]			
ADHD	0.249 [0.0007]			
		Control	<0.001	0.054 [0.04–0.068]
		Anxiety	<0.001	0.048 [0.032–0.065]
		depression	<0.001	0.046 [0.029–0.064]
		OCD	<0.001	0.046 [0.028–0.063]
autism	0.249 [0.0006]			
		Control	<0.001	0.054 [0.042–0.067]
		Anxiety	<0.001	0.048 [0.033–0.063]
		depression	<0.001	0.046 [0.03–0.063]
		OCD	<0.001	0.045 [0.03–0.061]
ptsd	0.248 [0.0006]			
		Control	<0.001	0.061 [0.048–0.074]
		Anxiety	<0.001	0.055 [0.04–0.07]
		depression	<0.001	0.053 [0.037–0.07]
		OCD	<0.001	0.052 [0.036–0.068]
schizophrenia	0.248 [0.0007]			
		Control	<0.001	0.067 [0.053–0.082]
		Anxiety	<0.001	0.061 [0.045–0.078]
		depression	<0.001	0.06 [0.042–0.077]
		OCD	<0.001	0.059 [0.042–0.076]
bipolar	0.243 [0.0008]			
		Control	<0.001	0.111 [0.097–0.127]
		Anxiety	<0.001	0.106 [0.088–0.123]
		depression	<0.001	0.104 [0.085–0.122]
		OCD	<0.001	0.103 [0.085–0.121]
		ADHD	<0.001	0.057 [0.038–0.076]
		autism	<0.001	0.058 [0.04–0.075]
		ptsd	<0.001	0.051 [0.033–0.068]
		schizophrenia	<0.001	0.044 [0.025–0.063]
DID	0.238 [0.001]			
		Control	<0.001	0.159 [0.14–0.177]
		Anxiety	<0.001	0.153 [0.132–0.173]
		depression	<0.001	0.151 [0.13–0.172]
		OCD	<0.001	0.15 [0.129–0.171]
		ADHD	<0.001	0.105 [0.083–0.126]
		autism	<0.001	0.105 [0.084–0.126]
		ptsd	<0.001	0.098 [0.077–0.119]
		schizophrenia	<0.001	0.091 [0.07–0.113]
		bipolar	<0.01	0.047 [0.025–0.07]

*Note*. Pairwise comparisons were performed on estimated marginal means and multiple comparisons were adjusted for using Tukey’s method.

## Discussion

We utilized NLP for the analysis of speech coherence, a pathological marker reflecting disorganized thinking, in posts gathered from the social media platform Reddit. NLP analysis revealed differences in the coherence of posts made in different forums related to mental health. Consistent with our hypothesis, coherence scores were lower in a forum on SZ than in a forum on depression. When analyzing coherence across a variety of psychopathology-related forum users, our analyses revealed the lowest coherence scores in DID and SZ forum users in dataset 1 and DID and BD forum users in dataset 2. In contrast, across both datasets, a relatively high level of coherence was detected for posts made by OCD, depression, and anxiety forum users. A control group reflective of the general Reddit user population showed the highest coherence scores.

Since the coherence score of a given text might be confounded by its length, emotional tone, and level of subjectivity, these measures were also extracted from posts and analyzed. The emotional valence of a post was negatively associated with the level of coherence. Additionally, for posts submitted to mental health subreddits, more personal/subjective stories were associated with a higher level of coherence. These findings align with previous research suggesting various prosocial consequences of coherent narration, such as increased social support, positive attitudes, and empathy toward narrators^65–67^. A central purpose of sharing personal experiences with others is the elicitation of social support^68^. Those users who share very negative personal experiences on Reddit probably do so because they seek peer support. Formulating a coherent post might aid them in this pursuit.

Our main analyses suggest an interesting trend whereby subreddits that showed the lowest coherence scores were geared toward disorders marked by more pronounced psychotic symptoms. In DID (>80% of patients^52^), BD (73.8% lifetime prevalence^49^), and PTSD (30-40% of combat veterans suffering from PTSD^53^), high rates of psychotic symptoms have been reported. In populations with autism spectrum disorder, a rate of 34.8% of comorbid SZ spectrum disorder diagnosis has been found^54^. In contrast, a heterogenous sample of subjects suffering from depressive and anxiety disorders (including PTSD), showed prevalence of psychotic symptoms of 27%^58^. In ADHD, no evidence of an increased risk of psychotic symptoms was found^57^. Only 14% of patients suffering from OCD were found to experience psychotic symptoms^55^. Speech incoherence may thus represent a psychopathological feature that varies in dependence on the degree of psychotic symptoms^59–61^. Consistent with this conclusion, symptoms of FTD, often equivalent to those found in SZ, have been found in BD and DID as well^48,50,51^.

Mental disorders are widely underrecognized and undertreated^1^. Diagnosis, prevention, and treatment programs based on large datasets obtained from online social media might offer promising solutions for challenges the mental healthcare field is facing^2^. Findings from laboratory studies indicate that speech coherence can be used to predict the onset of psychosis^37,38^. Our results provide the first evidence that incoherence may also be evidenced by social media posts. Future studies should investigate whether coherence may retain information about the presence, onset, or course of a disorder from the psychosis spectrum. Timely identification of high-risk individuals may allow for early-prevention and intervention programs that are more potent, less costly, and more widely available than interventions at advanced stages of the disorder such as inpatient treatment^69,70^. Because speech coherence analysis can be accompanied by automatic speech recognition technology without adverse effects on diagnostic accuracy^71^, such automated assessment pipelines might be a promising new diagnostic tool for future early prevention and treatment programs.

While several studies used NLP to examine linguistic features in social media posts made by subjects ostensibly suffering from psychosis^8,17–27^, this study explored the coherence of social media posts as an indicator of disorganization symptoms. A pronounced drawback of the present study is the lack of information on the users. Since the subreddits are open to all users irrespective of being “classified” as having mental health issues, the generalization of our findings to the clinical setting is not possible.

Additionally, with the rise and easy accessibility of generative large language models, the frequency of posts generated by machines (so-called “bots”) has increased. Consequently, the data quality of social media databases might become compromised and psychological investigations of real user behavior harder to perform. Information on user characteristics could help circumvent some of the limitations of our study. Additionally, information on the users’ clinical status or symptom severity might allow for more detailed analyses of psychosis-related incoherence in social media posts.

The coherence metric used here is related to a specific domain of FTD, namely tangentiality and derailment^31^. Given that many dimensions of FTD exist, future studies focusing on computational linguistic measures that correspond to other dimensions of FTD would be highly valuable. Notably, research on FTD and coherence has been predominantly performed on speech samples rather than written text^31,33^. Although written text is a more curated form of language production than speech, social media posts are arguably less curated than other written material. While our findings indicate that certain psychosis-related linguistic alterations are evidenced in written material, it still needs to be addressed how our findings correspond to data extracted from speech.

Lastly, we wish to emphasize that in order to use data from social media for clinical diagnosis and treatment, certain ethical and methodological considerations need to be taken into account. This is important to prevent users from being misidentified as being at high risk for a mental disorder or being assigned to an inappropriate treatment. Any sort of initial screening based on social media activity should be considered preliminary. Prior to the use of big social media data for clinical purposes, we need to ensure that collection methods are transparent, respecting user privacy rights, that findings are robust and interpreted in line with psychopathological models and that no harm is caused.

## Conclusion

We show that speech incoherence may be evidenced by social media posts. The most striking reduction in coherence emerged for forums on DID, SZ, and BD, the three disorders most commonly associated with psychotic and FTD symptomatology.

## Data Availability

Data are available from the first author upon reasonable request.

## Code availability

Code written for the purpose of this study is available from the first author upon reasonable request. For data extraction and preprocessing python (3.9.17), including the packages “numpy” (1.25.2), “pandas” (2.0.3), “tensorflow_hub” (0.15.0), “urlextract” (1.9.0), “textblob” (0.17.1) was used. For the statistical analysis R (4.3.1), including the packages “readxl” (1.4.3), “ggplot2” (3.4.3), “emmeans” (1.10.1), “car” (3.1-2), “stats” (4.3.1), “rstatix” (0.7.2), was used.
